# Letter to the editor: Livestock-associated meticillin-resistant *Staphylococcus aureus* (LA-MRSA), Austria, 2013

**DOI:** 10.2807/1560-7917.ES.2017.22.46.17-00758

**Published:** 2017-11-16

**Authors:** Werner Ruppitsch, Stefanie Monschein, Sarah Lepuschitz, Franz Allerberger, Burkhard Springer

**Affiliations:** 1 Institute for Medical Microbiology and Hygiene, Austrian Agency for Health and Food Safety (AGES), Graz, Austria

**Keywords:** Staphylococcus aureus, LA-MRSA, CC398, 'other' LA-MRSA

To the editor: In their article titled ‘Livestock-associated meticillin-resistant *Staphylococcus aureus* (LA-MRSA) among human MRSA isolates, European Union/European Economic Area countries, 2013’, Kinross et al. recently reported on the occurrence of LA-MRSA in humans [1]. The results were obtained by an ECDC initiated study documenting the identification of LA-MRSA (i.e. CC398 and ‘other’ LA-MRSA) in European Union/European Economic Area countries (EU/EEA) countries and the MRSA subtyping capacity and availability in EU/EEA national or regional reference laboratories. ECDC National Focal Points for Antimicrobial Resistance (AMR) were invited to designate a primary and alternate contact person with expertise in molecular surveillance of MRSA for public health purposes and with access to data for the survey in their respective countries; 27 of 30 EU/EEA countries responded to this request for data.

Data for Austria was missing in the report. We, the National Reference Laboratory (NRL) for coagulase-positive staphylococci, including *Staphylococcus aureus,* hereby report the missing data. In 2013, 250 human *S. aureus* isolates were obtained for typing: 18 isolates (7.2%) were of sequence type (ST)398 and belonged to five different spa types and six different cluster types (Table) [2]. Except for nine further human isolates of ST1 (spa type 127), no ‘other LA-MRSA’ was documented in 2013. All isolates were Panton–Valentine leukocidin (PVL)-negative.

**Table:** Spa-Types, geographical origin and type of sample of LA-MRSA isolates (n = 18) and human isolates of ST1 (spa type 127) (n = 9), Austrian National Reference Laboratory for coagulase-positive staphylococci, including *Staphylococcus aureus*, 2013

Sequence Type	Spa-Type	No. of isolates	Primary laboratory/province	Sample	CT (cgMLST)
398	t011	14	Hospital A Carinthia (n = 8) Hospital B Lower Austria (n = 4) Hospital C Vienna (n = 2)	Nose swab (n = 4) Urine (n = 1) Throat swab (n = 2) Perianal swab (n = 1) Wound swab (n = 5) Unknown (n = 1)	46, 1103, 395, 604, 98
	t034	1	Hospital A	Wound swab	1716
	t108	1	Hospital B	Unknown	Not done
	t571	1	Hospital D Vienna	Wound swab	Not done
	t3423	1	Hospital A	Urine	Not done
1	t127	9	Hospital A (n = 4) Hospital B (n = 4) Hospital C (n = 1)	Blood culture (n = 1) Wound swab (n = 4) Pleurocentesis fluid (n = 1) Bronchoalveolar lavage (n = 1) Unknown (n = 2)	Not done

cgMLST = core genome multilocus sequence typing; CT = cluster type; LA-MRSA: Livestock-associated meticillin-resistant *Staphylococcus aureus*.

Within Decision 2012/506/EU on case definitions for reporting communicable diseases, reporting of MRSA in the EU/EEA is included as a ‘Special health issue’ of ‘Antimicrobial resistance’ [3]. The Institute for Medical Microbiology and Hygiene, Austrian Agency for Health and Food Safety (AGES) in Graz was entrusted by the Austrian Ministry of Health with the tasks of a NRL for *Staphylococcus aureus* in 2007, and since then operates a sentinella system based on five hospitals. Our Austrian data with ST398 and ST1 (t127) accounting for 10.8% of 250 clinical MRSA-isolates tested, fit well with those 9.7% reported from the other nine NRLs that reported data from clinical samples only [1].

The increasing proportion of ST398 clonal complex strains, termed ‘LA-MRSA in isolates from human samples requires special attention. Much uncertainty remains about the origin and public health implications of LA-MRSA. AGES has started to survey the proportion of MRSA isolates from humans that were ST 398 in 2007 [4-6]. In the light of the increasing spread of LA-MRSA in Europe, Kinross et al. advocate that EU/EEA countries should consider periodically repeating this survey to monitor changes. They furthermore suggest that isolates from veterinary sources be included in such monitoring to systematically document potential reservoirs and transmission pathways to inform measures for prevention and control. We support such initiative and are pleased to contribute to this endeavour.
